# Intercellular transfer of multidrug resistance mediated by extracellular vesicles

**DOI:** 10.20517/cdr.2024.84

**Published:** 2024-09-21

**Authors:** Anxiang Yang, Hui Sun, Xiaokun Wang

**Affiliations:** ^1^Key Laboratory of Tropical Biological Resources of Ministry of Education, School of Pharmaceutical Sciences, Hainan University, Haikou 570228, Hainan, China.; ^2^School of Life and Health Sciences, Hainan University, Haikou 570228, Hainan, China.

**Keywords:** Multidrug resistance, extracellular vesicles, intercellular transfer, intercellular communication, ABC transporter

## Abstract

Multidrug resistance (MDR) poses a formidable obstacle in cancer treatment, enabling cancer cells to evade the cytotoxic effects of chemotherapeutic drugs through various mechanisms. These mechanisms include intrinsic resistance, which is present prior to treatment, and acquired resistance, which develops after exposure to chemotherapy agents. Small membrane-bound vesicles, known as extracellular vesicles (EVs), are crucial in intercellular signaling as they transport bioactive molecules that can modify the characteristics and functions of recipient cells. Recent research highlights EVs as pivotal players in fostering drug resistance. This review focuses on the intercellular transfer of MDR from donor cells to susceptible recipient cells through specific cargo in EVs, such as ATP-binding cassette (ABC) transporter proteins, nucleic acids, and other regulatory factors. Additionally, the features of intercellular communication mediated by EVs are also discussed. Gaining insight into these mechanisms is essential for developing strategies to counteract resistance and improve the effectiveness of cancer treatments.

## INTRODUCTION

Extracellular vesicles (EVs) are a recently defined collective term that refers to various types of nano-sized, lipid bilayer-enclosed vesicles^[1]^. These nanovesicles, once regarded as “cellular waste products”, are now recognized as key regulators of various biological functions and intercellular communication^[2]^. EVs facilitate cellular information exchange by carrying a diverse array of soluble and insoluble signaling molecules, structural proteins, nucleic acids, and lipids. The molecular cargo and biological functions of EVs are primarily determined by their originating cells^[3]^. In addition to cell-to-cell contact, EVs can travel through body fluids, delivering their contents to distant target cells, thereby mediating systemic cellular communication *in vivo*^[3-5]^.

Despite the numerous challenges in the isolation and characterization of EVs, it is increasingly evident that EVs containing functional proteins, nucleic acids, and other bioactive molecules can transfer to recipient cells and modulate their behavior. Recent studies have identified a functional role for EVs in communication between cancer and immune cells. These properties make EVs a promising tool for cancer immunotherapy and diagnostics. EVs are implicated in several aspects of tumor progression, including altering the tumor microenvironment (TME), promoting tumor growth, and facilitating angiogenesis and metastasis^[6]^.

Multidrug resistance (MDR) remains a significant challenge in cancer treatment. Cancer cells can evade the cytotoxic effects of chemotherapeutic agents through intrinsic or acquired resistance mechanisms. Emerging evidence indicates that EVs play a crucial role in developing drug resistance. These vesicles can disseminate resistance to chemotherapy, radiotherapy, targeted therapy, and immunotherapy by transferring specific proteins, nucleic acids, survival signaling molecules, and genetic material to cancer cells^[7]^. Moreover, EVs contribute to therapy resistance by influencing the interactions between cancer cells and non-cancer cells within the TME^[8]^. For instance, tumor-derived exosomes can reprogram cancer-associated fibroblasts (CAFs) to create an acidic TME, enhancing resistance to platinum-based drugs in non-small cell lung cancer (NSCLC)^[9]^. Recently, EVs have emerged as ideal candidates for drug development and delivery due to their stability, biocompatibility, and targeting capabilities^[10]^. Engineered and synthetic vesicles hold potential as drug delivery vehicles to overcome cancer drug resistance and boost therapeutic efficacy. Understanding the role and mechanisms of EVs in MDR development can help enhance chemotherapy effectiveness and maximize the therapeutic benefits of EVs in cancer treatment. This review briefly summarizes the biogenesis of EVs and highlights key advances in intercellular communication mediated by EVs. We emphasize the “non-genetic” transfer of MDR via specific mediators in EVs, such as ATP-binding cassette (ABC) transporter proteins, nucleic acids, and other regulatory factors.

## EVS

EVs are a heterogeneous population that includes exosomes, microvesicles, and apoptotic bodies, which are released by various cell types. These membranous vesicles are generally distinguished by their size and biogenesis^[11]^. Exosomes, which range from 30 to 150 nm in diameter, are produced through the classical endosome-multivesicular body (MVB) pathway, followed by fusion with the plasma membrane^[12]^. Microvesicles, ranging from 150 to 1,000 nm in diameter, are shed directly from the plasma membrane. Apoptotic vesicles, even larger vesicles with diameters ranging from 500 to 2,000 nm, form during programmed cell death and are characterized by their substantial amounts of RNA, distinguishing them from other microvesicles^[13-15]^. However, recent studies have also identified smaller apoptotic exosomes (under 500 nm) that differ in size, content, properties, and function from traditional apoptotic vesicles^[16-18]^. New EV subtypes have also been discovered, such as oncosomes, which are even larger (1 to 10 μm) and are exclusively shed from the plasma membrane of cancer cells^[6]^. EV subtypes often share similarities; for example, some microvesicles are less than 150 nm in diameter. Most EVs reported in published studies are actually a mixture of vesicles, primarily because the focus has often been on their potential biological roles rather than their origins. Despite recent advances in understanding EVs, it remains challenging to isolate specific EV classes due to technical limitations in purification and isolation. Furthermore, assigning EVs to particular biogenesis pathways is complicated, as there are no widely recognized specific markers for EV subtypes. The International Society for Extracellular Vesicles (ISEV) has urged authors to use more precise terms to define EVs according to their physical characteristics, biochemical composition, and cell of origin^[13]^.

EVs contain a variety of biologically active molecules, including proteins, nucleic acids, and lipids. The proteins enclosed in EVs include extracellular matrix (ECM) components, membrane proteins, and nuclear proteins. Several tetra-transmembrane proteins (e.g., CD9, CD37, CD81, CD82, and CD63) are particularly abundant in exosomes and microvesicles^[19-21]^. CD63 and the cytosolic protein heat shock 70kDa protein (HSP70) are frequently used as exosome marker proteins^[22,23]^. Cytosolic metabolites and nucleic acids (e.g., DNA, noncoding RNA, and mRNA) are also found in exosomes^[24,25]^. On the outer surface, exosomes feature glycan crowns attached to lipids and proteins, with underlying phospholipids, cholesterol, ceramides, glycerides, and sphingolipids playing key roles in EV biogenesis, uptake, and their functional effects on recipient cells^[26-28]^. The cargo composition and abundance of EVs vary depending on the type of donor cells, metabolic regulation, and disease states^[29]^. The process of EV cargo selection is precisely and dynamically regulated. Cargo-driven biogenesis largely contributes to EV heterogeneity, and in some cases, specific cargoes unique to certain cell types can serve as biomarkers for tissue or disease diagnosis^[29]^. The endosomal sorting complex required for transport (ESCRT) machinery, which is crucial for membrane shaping and scission, is a major regulator of exosome biogenesis and a key determinant of their functional properties^[30]^. The biogenesis of microvesicles involves multiple molecular rearrangements in the plasma membrane, including those involving lipids, cytoskeletal elements, and their regulators^[30]^. For instance, depleting membrane cholesterol pharmacologically impairs microvesicle shedding in activated neutrophils^[31]^. The “Warburg effect”, a hallmark of cancer metabolism, is associated with the biogenesis of oncosomes^[32]^. In the release process of EVs, Rab family proteins and soluble N‐ethylmaleimide‐sensitive fusion attachment protein receptors (SNAREs) are important regulators^[33,34]^. Additionally, factors such as low pH, hypoxia, and increased external pressure can promote EV release^[8]^.

Once in the extracellular environment, EVs can enter recipient cells by endocytosis or direct fusion with the target cell membrane^[35]^. The recipient cells determine the route of EV entry and their subsequent fate^[36]^. The specific composition of EVs also affects their fate. EVs deliver their contents to elicit phenotypic and functional changes in recipient cells through three main mechanisms: (1) Direct activation of target cells via interactions between surface ligands on the EVs and receptors on the target cells; (2) Transfer of activated receptors; and (3) Genetic reprogramming through the delivery and translation of mRNAs or post-translational modifications^[37]^.

## INTERCELLULAR COMMUNICATION OF EVS

EVs are secreted by a diverse range of cell types, including tumor cells, neurons, epithelial cells, and various immune cells such as dendritic cells, T cells, B cells, and mast cells^[38]^. Initially identified as “cellular dust”, EVs were once believed to serve merely as a means for cells to eliminate unwanted components. However, accumulating research has revealed that EVs are actively involved in transferring information between cells, significantly impacting cellular functions and facilitating intercellular communication.

### Intercellular communication via EVs among tumor cells

Growing evidence shows the intercellular transfer of EVs between tumor cells. For example, glioma cells that secrete EVs containing EGFRvIII can fuse with the plasma membranes of other glioma cells lacking EGFRvIII, thereby transferring oncogenic activity^[39]^. The transfer of EGFR-rich EVs also occurs from highly to poorly metastatic nasopharyngeal carcinoma cells^[40]^. This transfer of oncogenic signaling components from one cell to another underscores the important role of EVs in cancer. Recent evidence indicates that mRNA and miRNA transferred by EVs from cancer cells can influence tumorigenesis, drug resistance, and tumor progression^[41,42]^. For example, exosomes from cisplatin (DDP)-resistant cancer cells have been shown to transfer miR-100-5p to recipient lung cancer cells, altering their resistance to DDP^[43]^. Exosomes released from pancreatic ductal adenocarcinoma (PDAC) cells with long-term exposure to gemcitabine delivered miR-155 to other PDAC cells, inducing chemoresistance^[44]^. Additionally, exosomes from gemcitabine-resistant A549 cells carrying miR-222-3p have been found to enhance proliferation, gemcitabine resistance, migration, and invasion in sensitive parental cells^[45]^.

### Intercellular communication via EVs between non-tumor cells

Intercellular transfers of EVs in non-tumor cells were also reported. For instance, exosomes released by CD4+ T cells can suppress the responses of CD8+ cytotoxic T lymphocytes and thereby inhibit antitumor immunity^[46]^. Ying *et al.* demonstrated that exosomes containing miRNA secreted by M1 macrophages can be transferred to adipocytes, causing glucose intolerance and insulin resistance^[47]^. The transfer of multiple molecules, such as miRNAs, gangliosides, and proteins, by EVs has been found to affect neurodegeneration^[48]^. Morel *et al.* characterized a new exosome-mediated transfer of miRNA from neurons to astrocytes^[49]^. Intercellular communication by EVs between various types of cardiac cells has been widely reported^[50]^. EVs originating from leukocytes, erythrocytes, smooth muscle cells, and endothelial cells can affect processes such as inflammation, thrombosis, neoangiogenesis, cell survival, and endothelial homeostasis, thus playing a role in the initiation and progression of atherosclerosis^[51,52]^.

### Intercellular communication via EVs between tumor cells and non-tumor cells

Intercellular communication of EVs also occurs between cells of different origins. For instance, EVs are transferred between immune cells and tumor cells. Exosomes from hypoxic human epithelial ovarian cancer cells are transferred to macrophages, resulting in the differentiation of macrophages towards an anti-inflammatory M2 phenotype^[53]^. Colorectal cancer-derived exosomes containing miR-934 promote M2 macrophage polarization and induce pre-metastatic niche formation^[54]^. Gastric cancer-derived exosomes can be internalized by intrahepatic macrophages, causing M2-like polarization that accelerates liver metastasis of gastric cancer^[55]^. Accumulating evidence demonstrates that tumor-derived exosomes can transfer to dendritic cells and interfere with their maturation, differentiation, and function^[56]^. Zhang *et al.* showed that tumor cell-derived exosomes containing circUHRF1 induce exhaustion in natural killer cells and drive resistance against anti-PD1 immunotherapy^[57]^.

Communication between cancer cells and CAFs mediated by EVs has also been reported. Malignant mesothelioma-derived exosomes are suggested to facilitate the migratory capacity of CAFs^[58]^. Zhao *et al.* revealed that CAF-derived exosomes transport essential nutrients to cancer cells and modulate cancer cell metabolism^[59]^. Tumor-derived EVs interact with endothelial cells to exhibit proangiogenic activities by delivering activated EGFR^[60]^.

## INTERCELLULAR TRANSFER OF MDR BY EVS

MDR is characterized by the cross-resistance of cancer cells to a range of chemotherapeutic drugs that have distinct structures and mechanisms of action^[61]^. This resistance significantly contributes to the failure of chemotherapy and is commonly associated with the recurrence of tumors. MDR can be categorized into two types: intrinsic resistance and acquired resistance. The former refers to pre-existence before the use of chemotherapeutic drugs, and the latter arises by multiple mechanisms after exposure to chemotherapeutics. Several potential mechanisms underlying MDR have been identified in cancer cells, including reduced drug uptake, increased drug efflux by the ABC transporter family, regulation by cancer stem cells, altered DNA damage response and repair, induction of hypoxia, altered drug targets and sequestration of anticancer drugs in intracellular organelles^[61-67]^. Among these mechanisms, the high expression of the ABC transporter family is the predominant molecular mechanism underlying MDR in cancer cells. Recent studies have revealed a novel mechanism responsible for MDR, in which EVs directly transport various proteins, including ABC transporters and proteins related to tumor survival and DNA repair, to recipient cells [Table 1]. Additionally, EVs can transport RNAs, including miRNAs and long noncoding RNAs (lncRNAs), from donor cells to recipient cells, which can lead to the development of drug resistance^[68]^ [Table 1]. Furthermore, the intercellular transfer of EVs can reshape the TME, creating conditions that favor therapy resistance^[69,70]^.

**Table 1:** Diverse cargo types in EV-mediated transfer of MDR

**Cargo type**	**Molecules**	**Drug resistance**	**Model**	**Refs.**
Drug efflux pumps	ABCB1	Colchicine	Human neuroblastoma cell line BE(2)-C, and human breast adenocarcinoma cell line MCF-7	[78]
		Carboplatine, taxol	Primary stromal cells and human ovarian cancer cell line OVCAR3	[79]
		Daunorubicin	Human acute lymphoblastic leukemia cell line CCRF-CEM	[80]
		Docetaxel	Human prostate cancer cell lines 22Rv1, DU145 and LNCap	[81]
		NA	Human acute lymphoblastic leukaemia cell line CCRF-CEM, and human breast adenocarcinoma cell line MCF-7	[82]
		Docetaxel	Human breast adenocarcinoma cell line MCF-7	[83]
		NA	Human breast adenocarcinoma cell line MCF-7	[84]
		NA	Human bladder cancer cell line BIU-87	[86]
		Doxorubicin	Human bladder cancer cell line BIU-87	[87]
		Adriamycin, paclitaxel	Human ovarian cancer cell line A2780	[88]
		Doxorubicin	Human osteosarcoma cell line MG-63	[89]
		Doxorubicin	Human oral epidermoid carcinoma cell line KB, and human colon carcinoma cell line S1	[90]
	ABCC1	NA	Human acute lymphoblastic leukaemia cell CCRF-CEM, and human breast adenocarcinoma cell line MCF7	[94]
		Daunorubicin	Human acute promyelocytic leukemia cell line HL60	[95]
Nucleic acids	miR-27a and miR-326	NA	Human acute lymphoblastic leukemia cell line CCRF-CEM, and human breast adenocarcinoma cell line MCF-7	[106]
	miR-100, miR-222, and miR-30a	NA	Human breast adenocarcinoma cell line MCF-7	[7]
	miR‐21‐5p	DDP	Human ovarian cancer cell line SKOV3	[107]
	miR-301b-3p	DDP/VCR	Human gastric cancer cells	[108]
	miR-221/222	Tamoxifen	Human breast adenocarcinoma cell line MCF-7	[109]
Other regulators	TrpC5	Adriamycin	Human breast adenocarcinoma cell line MCF-7	[113]
	EGFR	Osimertinib	Human NSCLC cell lines H460, A549, H1299, H1975, and PC9	[114]
	PDGFRβ	PLX4720	Human melanoma cell line LM-MEL-64	[115]
	HGF	Sorafenib	Human hepatocellular carcinoma cell line SMMC-7721, MHCC-97H, and MHCC-97 L	[117]
	Survivin	Paclitaxel	Human breast cancer cell line MDA-MB-231	[119]

EV: Extracellular vesicle; MDR: multidrug resistance; DDP: cisplatin; TrpC5: transient receptor potential channel 5; NSCLC: non-small cell lung cancer; PDGFRβ: platelet-derived growth factor receptor-beta; HGF: hepatocyte growth factor.

### Transfer of ABC transporter proteins by EVs

ABC transporters are a family of membrane proteins that utilize ATP hydrolysis to transport a diverse array of substrates, including ions and macromolecules, across extra- and intercellular membranes. The human ABC transporter family includes 48 members, classified into seven subfamilies (A to G) based on sequence homology, domain organization, and phylogenetic analysis^[71]^. A canonical ABC transporter consists of two highly variable transmembrane domains (TMDs) embedded in the membrane bilayer and two highly conserved nucleotide-binding domains (NBDs) located in the cytoplasm. The TMDs provide the specificity for the substrate and the NBDs transfer energy to transport the substrate across the membrane^[71]^. Based on their function, ABC transporters are divided into three classes: exporters, importers, and non-transporters. Although most ABC transporters are exporters, ABCA4 and ABCD4 function as importers^[72,73]^. The ABCE and ABCF families do not function as transporters but act as regulators of mRNA translation^[74]^. Over the past decade, at least 13 ABC transporters have been implicated in the development of MDR *in vitro*. The high expression of ABC transporters, particularly ABCB1, ABCC1, and breast cancer resistance protein (BCRP), is a widely recognized molecular mechanism that contributes to MDR in cancer cells. More than 50% of cancers exhibiting an MDR phenotype have been found to express these ABC transporters constitutively or inducibly, enabling them to expel chemotherapeutic drugs and reduce intracellular drug concentrations^[75]^.

#### ABCB1

ABCB1, also known as P-glycoprotein (P-gp), is the first and most extensively studied member of the ABC transporter family. It is closely associated with the MDR phenotype by actively exporting a broad spectrum of chemotherapeutic agents from cancer cells. In mammals, particularly in humans, ABCB1 is prominently expressed in specific tissues such as the intestine, liver, kidney, and brain, where it plays a key role in drug transport and detoxification processes^[76]^. ABCB1 consists of two homologous NBDs and two homologous TMDs. The NBDs create a large pocket essential for ATP binding and hydrolysis, which is critical for ATPase activity and energy generation. Meanwhile, the TMDs form a flexible channel with 12 transmembrane helices responsible for substrate recognition and transportation^[76]^. Clinical studies have demonstrated that ABCB1 is overexpressed in various malignant tumor cells and is associated with chemotherapy efficacy and cancer progression^[77]^. The increased expression of ABCB1 in cancer cells enhances the efflux of various structurally and mechanistically different anticancer drugs, such as paclitaxel, docetaxel, vincristine, doxorubicin, imatinib, and nilotinib, leading to decreased intracellular drug levels and subsequent chemoresistance.

The intercellular transfer of ABCB1 via EVs has been supported by extensive experimental evidence. Levchenko *et al.* first reported in 2005 that functional ABCB1 can transfer from drug-resistant to drug-sensitive cancer cells both *in vitro* and *in vivo*, resulting in increased drug resistance without the expression of the *ABCB1* gene^[78]^. This transfer was mediated by relatively large EVs (> 0.8 μm in diameter) or required cell-to-cell contact, but not by small EVs (below 0.2-μm in diameter)^[78]^. This study also demonstrated that the functional transfer of ABCB1 occurred across different tumor cell types, suggesting a broad mechanism for the dissemination of MDR^[78]^. Subsequent studies have further supported the intercellular transfer of ABCB1. For instance, Rafii *et al.* found that ovarian cancer cells gain chemoresistance through the transfer of functional ABCB1 proteins from an original type of stromal cells^[79]^. In acute lymphoblastic leukemia cells, ABCB1 proteins carried by EVs from drug-resistant cells were found to transfer into drug-sensitive recipient cells, leading to an MDR phenotype within as early as 2 h^[80]^. The rapid transfer of ABCB1 by EVs and the swift acquisition of drug resistance in recipient cells within two hours suggest that this process likely involves the direct transfer of exogenous ABCB1 protein from donor cells, rather than the upregulation of *ABCB1* gene expression in recipient cells. Corcoran *et al.* demonstrated that exosomes derived from docetaxel-resistant prostate cancer cells notably enhanced docetaxel resistance in previously sensitive recipient cells, partly attributed to the transfer of ABCB1^[81]^. Similarly, Jaiswal *et al.* observed a stable transfer of ABCB1 mediated by microparticles (MPs) in both *in vitro* and *in vivo* settings^[82]^. Comparable transfer of drug resistance and ABCB1 protein by EVs have been reported in breast cancer, bladder cancer, and ovarian cancer cells^[83-88]^. Additionally, drug-resistant osteosarcoma cells have been shown to transfer exosomes containing both ABCB1 protein and mRNA, thereby conferring doxorubicin resistance to sensitive cells^[89]^. Our recent study indicates that in the presence of chemotherapeutic drugs, the intercellular transfer of ABCB1 is enhanced through the stimulated secretion and recycling of ABCB1-enriched EVs, suggesting a higher efficiency of this transfer under selective pressure from chemotherapeutic agents^[90]^.

Jaiswal *et al.* found that the MDR phenotype acquired by recipient cells remained stable for at least 5 days *in vitro*, even in the absence of selecting pressure^[91]^. In an MCF-7 tumor xenograft model, the MDR phenotype can be rapidly acquired by drug-sensitive cancer cells within 24 h and can persist for at least 2 weeks *in vivo*^[82]^. The MDR phenotype transferred by EVs protected sensitive tumor cells for up to 4 months, facilitating the development of intrinsic ABCB1-mediated resistance^[78]^. However, the transfer of ABCB1 protein via EVs alone does not fully account for the long-term persistence of this MDR phenotype, especially considering that the half-life of ABCB1 is approximately 14 to 17 h. One plausible explanation is that other cargo, such as miRNAs, is delivered to recipient cells alongside ABCB1 by EVs.

#### ABCC1

ABCC1, also known as multidrug resistance-associated protein 1 (MRP1), plays a significant role in the recognition and efflux of both hydrophobic and hydrophilic antineoplastic agents, resulting in decreased drug accumulation within tumor cells and contributing to the development of MDR. Elevated levels of ABCC1 are strongly associated with poor prognosis and decreased survival across multiple cancer types^[92,93]^.

Emerging evidence suggests that ABCC1 is also involved in the transfer of MDR between cells via EVs. For example, Lu *et al.* reported the intercellular transfer of functional ABCC1 via MPs^[94]^. Lymphoblastic leukemia cells that overexpress ABCC1 spontaneously shed MPs containing ABCC1, which can then transfer to drug-sensitive cells within 12 h of co-culture^[94]^. Another study revealed that chemoresistance can be transmitted from drug-resistant leukemia cells to their parental counterparts via EVs, likely due to the direct transfer of ABCC1^[95]^.

#### ABCG2

ABCG2, also known as BCRP, is a “half-transporter” featuring a single NBD and a single TMD^[96]^. Unlike other ABC transporters, ABCG2 functions as a homodimer, comprising two identical subunits. The dimerization of ABCG2 is essential for its transport activity, as the NBDs must dimerize to form a functional structure necessary for ATP binding and hydrolysis^[97]^. ABCG2 is highly expressed in the placenta, blood-brain barrier, liver, and intestine, and is notably prevalent in various stem cells, including hematopoietic stem cells. Its expression is associated with the “side population” phenotype, which is associated with stemness and drug efflux capability^[98]^. ABCG2 is also a well-recognized transporter involved in MDR across various tumor types. It has been reported to transport several anticancer drugs, including mitoxantrone, topotecan, and irinotecan. The ability of ABCG2 to expel a wide range of drugs from cancer cells enables these cells to evade the cytotoxic effects of chemotherapy. Elevated ABCG2 expression has been implicated in cancer progression and poor clinical outcomes^[99]^.

Recent studies have emphasized the role of ABCG2 in the function and composition of EVs. ABCG2 is abundantly expressed on the membrane of EVs secreted by cancer cells. These EVs can encapsulate and export chemotherapeutic drugs out of the cell, reducing intracellular drug concentrations and contributing to enhanced drug resistance. Ifergan *et al.* suggest that mitoxantrone resistance may result from its entrapment within the vesicular lumen of EVs mediated by overexpressed ABCG2 on the EV membrane^[100]^. They also suggest that the levels of MDR mediated by ABCG2-rich EVs can be assessed by measuring the ABCG2-dependent intravesicular concentration of riboflavin in EVs^[101]^. Additionally, ABCG2-enriched EVs can concentrate and sequester various antitumor drugs, increasing the resistance of EVs-forming breast cancer cells to topotecan, imidazoacridinones, and methotrexate^[102]^. This proposed a novel MDR mechanism where ABC transporters are specifically sorted to the EV membrane, actively sequestering anticancer drugs inside EVs and leading to significant chemoresistance^[102]^. Moreover, the PI3K-Akt signaling pathway can inhibit the capacity of EVs to sequester anticancer drugs by gradually retracting ABCG2 from the EV membrane into the cytoplasm^[103]^. As a crucial regulator of ABCG2 localization, suppression of the PI3K-Akt signaling pathway decreases the sorting of ABCG2 to EVs, eventually reducing EV production and reversing MDR. Further studies suggest that the size and number of EVs gradually decrease due to the retention of intracellular ABCG2^[103]^. These findings have provided a wealth of information on the role of ABCG2 in the biogenesis of EVs. However, to date, there is no direct evidence suggesting the spread of MDR through the intercellular transfer of ABCG2-containing EVs, which requires further investigation.

### Transfer of nucleic acids by EVs

EVs derived from cancer cells carry various types of genetic material, including mRNA, lncRNAs, ribosomal RNAs (rRNAs), and small nuclear RNAs (snRNAs), all of which are functionally active^[104]^. Recent research suggests that EVs play a role in the dissemination of MDR through intercellular nucleic acid exchange, particularly via miRNAs^[105]^. miRNAs are single-stranded, noncoding RNA that regulate specific mRNA targets and are implicated in the development of chemoresistance by influencing multiple genes. The aberrant expression of multiple miRNAs has been implicated in resistance to various anticancer drugs such as topotecan, doxorubicin, methotrexate, docetaxel, and DDP. Earlier studies emphasized the crucial role of miRNAs within EVs in the dissemination of MDR. For example, Jaiswal *et al.* provided evidence that transporter transcripts and regulatory nucleic acids (miRNAs) can be transferred via EVs isolated from ABCB1-overexpressing multidrug-resistant leukemia and breast cancer cells. These EVs can alter the transcriptional environment of recipient cells, thereby reflecting the donor MDR phenotype^[106]^. Recent studies have pinpointed specific miRNAs within EVs that contribute to MDR dissemination. Chen *et al.* demonstrated that EVs from drug-resistant breast cancer cells convey chemoresistance in an RNA-dependent manner, with microRNAs such as miR-100, miR-222, and miR-30a being transferred to sensitive recipient cells. Further transfection experiments with miRNA mimics indicated that miR-222 mimics could regulate drug resistance by targeting PTEN, with miR-222-rich EVs altering PTEN expression in drug-sensitive breast cancer cells^[7]^. Similarly, exosomal miR-21-5p has been shown to influence cancer cell chemosensitivity by targeting pyruvate dehydrogenase E1 subunit α 1 (PDHA1). In DDP-resistant SKOV3 cells and tumor tissues, miR-21-5p expression was upregulated, while PDHA1 levels were significantly decreased. Exosomes from DDP-resistant ovarian cancer cells increased the resistance of sensitive cells to DDP and promoted cell viability by targeting PDHA1 with exosomal miR-21-5p^[107]^. Zhu *et al.* reported that EVs carrying miR-301b-3p from mesenchymal stem cells (MSCs) induced MDR in gastric cancer cells by suppressing thioredoxin-interacting protein (TXNIP)^[108]^. Inhibition of miR-301b-3p or upregulation of TXNIP increased the chemosensitivity of DDP/VCR-resistant gastric cancer cells and reversed malignant behaviors. Another study suggests that EVs carrying miR-221/222 transmit tamoxifen resistance to drug-sensitive breast cancer cells^[109]^. Exosomes from tamoxifen-resistant breast cancer cells can enter tamoxifen-sensitive cells and release miR-221/222, leading to enhanced resistance by reducing the expression of p27 and ERα, targets of miR-221/222^[109]^.

EVs are emerging as effective and promising vehicles for drug delivery due to their natural properties of low immunogenicity and high biocompatibility^[110]^. EVs can protect miRNAs from degradation by RNases, unlike naked miRNAs, opening new avenues for overcoming MDR. For instance, miR-9 regulates the expression of the drug efflux transporter ABCB1. Munoz *et al.* suggested that microvesicles are the primary carriers of anti-miR-9 from MSCs to glioblastoma multiforme cells, reversing chemoresistance by downregulating ABCB1^[111]^. Similarly, paclitaxel-sensitive NPC cells have been reported to deliver miR-183-5p to paclitaxel-resistant NPC cells via EVs, thereby suppressing paclitaxel resistance by negatively regulating ABCB1^[112]^.

### Transfer of other regulators by EVs

In addition to ABC transporters, other proteins transported via EVs may also significantly contribute to the development of MDR. Ma *et al.* found that transient receptor potential channel 5 (TrpC5) can be delivered to sensitive cancer cells via EVs derived from drug-resistant breast cancer cells. This transfer enables the recipient cells to acquire the Ca^2+^-permeable channel, which in turn stimulates P-gp expression through a Ca^2+^- and NFATc3-mediated mechanism, ultimately conferring chemoresistance to the previously sensitive cells^[113]^. A recent study suggested that exosomes can transfer wild-type EGFR, which, upon internalization by EGFR-mutated cancer cells, activates downstream PI3K/AKT and MAPK signaling pathways, thereby conferring osimertinib resistance to EGFR-mutated sensitive cancer cells both *in vitro* and *in vivo*^[114]^. Platelet-derived growth factor receptor-beta (PDGFRβ), a critical factor in resistance, is enriched in EVs released by melanoma cells that are resistant to the BRAF inhibitor PLX4720. Upon transfer to recipient melanoma cells, PDGFRβ triggers a dose-dependent activation of the PI3K/AKT signaling pathway, enabling these cells to circumvent BRAF inhibition^[115]^. Ji *et al.* found that exosomes originating from MSCs induce resistance to 5-fluorouracil in gastric cancer cells, with elevated expression of multiple drug-resistance-associated proteins, including ABCB1, ABCC1, and LRP1^[116]^. Interestingly, MSC exosome-delivered proteins, rather than ABCB1 alone, played a dominant role in acquired resistance^[116]^.

EVs also transport molecules that promote cell survival, including prosurvival factors and proteins involved in apoptosis regulation. By improving the viability of recipient cells and decreasing their susceptibility to apoptosis, these components contribute to the development of MDR. For instance, in hepatocellular carcinoma (HCC) cell lines, EVs carrying hepatocyte growth factor (HGF) have been shown to induce resistance to Sorafenib both *in vitro* and *in vivo*. This resistance is mediated by the activation of the HGF/c-MET/PI3K/AKT signaling pathway, which is crucial for cell survival and drug resistance^[117]^. Additionally, EVs from apoptosis-resistant acute myeloid leukemia (AML) blasts contain proteins that regulate apoptosis and impart a drug-resistant phenotype to primary AML cells that are initially sensitive to apoptosis^[118]^. Survivin, a prosurvival protein and a member of the inhibitors of apoptosis (IAP) family, plays a vital role in preventing cell death and regulating mitosis. Kreger *et al.* reported that treatment of highly aggressive MDA-MB-231 breast cancer cells with paclitaxel stimulates the release of EVs rich in survivin. These survivin-enriched EVs significantly enhance the survival of serum-starved and PTX-treated fibroblasts and SKBR3 breast cancer cells^[119]^.

Beyond the direct transfer of bioactive molecules, EVs also reshaped the TME, facilitating the transmission of MDR. Cell-to-cell communication within the TME occurs through direct contact or the exchange of bioactive molecules via EVs. EVs are recognized as key regulators of tumor dynamics. Lopes-Rodrigues *et al.* identified metabolic alterations in ABCB1-overexpressing cells, with EVs from MDR cells altering metabolic profiles in drug-sensitive cancer cells and thereby promoting MDR transfer^[120]^. Notably, EV-mediated communication between tumor cells and macrophages or fibroblasts is well-documented in TME modulation^[121]^. Fibroblasts are crucial for producing the ECM and facilitating wound healing^[122]^. CAFs, which are predominant in the TME, exhibit intrinsic resistance to gemcitabine. Richards *et al.* reported that gemcitabine exposure significantly increases EV secretion from CAFs, promoting proliferation and drug resistance in recipient pancreatic adenocarcinoma epithelial cells via upregulation of Snail^[123]^. These findings underscore the critical role of EV-mediated TME reshaping in the development of MDR.

## STRATEGIES TO OVERCOME EV-MEDIATED DRUG RESISTANCE

Given the pivotal role of EVs in MDR, it is crucial to develop strategies to counteract this form of drug resistance. One direct approach involves inhibiting the production and release of EVs from drug-resistant cells. Various pharmacological agents have been identified that can interfere with EV formation and release, thereby enhancing the efficacy of anticancer treatments. GW4869, a known inhibitor of neutral sphingomyelinase, is commonly used to impede EV production and diminish EV release. Research by Hekmatirad *et al.* showed that GW4869 effectively prevents the efflux of PEGylated liposomal doxorubicin (PLD) and increases U937 cell sensitivity to PLD’s cytotoxic effects^[124]^. Similarly, blocking EV release from CAFs with GW4869 reduces the transfer of chemoresistance and decreases the survival of pancreatic cancer cells^[123]^. Moreover, extracellular signal-regulated kinase (ERK) has been linked to EV release. Using the MEK inhibitor U0126 to block ERK activation sensitizes gemcitabine-resistant pancreatic cells to gemcitabine, both *in vitro* and *in vivo*^[125]^. Federici *et al.* found that Lansoprazole, a proton pump inhibitor, reduces overall EV release from tumor cells, thereby enhancing sensitivity to DDP^[126]^. Additionally, Rab GTPases, which are critical for EV trafficking and release, can be targeted. For example, siRNA-mediated inhibition of Rab27a reduces EV release and significantly decreases miR-155-induced gemcitabine resistance in pancreatic cancer cells^[44]^.

Another approach involves preventing the uptake of EVs by recipient cells. Disrupting the interaction between EVs and their target cells can limit the transfer of MDR-associated factors. Heparin, a glycosaminoglycan, can inhibit EV binding to cell surface proteins, thereby reducing EV uptake. Studies have demonstrated that heparin treatment significantly decreases the transfer of EVs containing EGFRvIII mRNA between tumor cells^[127]^. EVs are frequently internalized through macropinocytosis, and inhibitors of this process, such as 5-(N-ethyl-N-isopropyl) amiloride (EIPA), can block EV uptake and restore EGFR-TKI sensitivity in PC-9 cells^[128]^.

Despite the promising potential of these strategies to overcome EV-mediated MDR, several challenges must be addressed to ensure their clinical effectiveness. The heterogeneity of EVs in size, content, and function complicates the development of universal targeting strategies. Additionally, targeting EVs may affect normal physiological processes, as EVs are produced by both cancerous and healthy cells and play crucial roles in immune modulation. Inhibiting EVs might inadvertently disrupt beneficial immune responses, potentially leading to tumor progression or immune-related side effects. Furthermore, designing effective therapies to block EV-mediated MDR presents technical difficulties due to the need for precise molecular targeting. Future research should aim to enhance our understanding of EV biology, refine targeting methods, and address potential resistance mechanisms.

## CONCLUSION

Tumor MDR represents a significant challenge in cancer therapy, often arising from complex genetic alterations induced by drug exposure. However, recent findings underscore a novel non-genetic mechanism in which EVs mediate the transfer of drug resistance between cells. Specifically, EVs from drug-resistant tumor cells can horizontally transfer various components, such as drug-efflux pumps (e.g., ABCB1, ABCC1), miRNAs (e.g., miR-21-5p, miR-222), and regulatory proteins (e.g., TrpC5, EGFR), to drug-sensitive cells, thereby spreading a drug-resistant phenotype. Furthermore, EV-mediated intercellular communication within the TME plays a crucial role in MDR propagation. These insights into non-genetic mechanisms of MDR not only provide opportunities for developing novel therapeutic strategies to overcome MDR but also hold promise for identifying biomarkers of drug resistance. Despite remarkable progress, the intercellular transfer of MDR via EVs remains poorly understood. Key aspects requiring further investigation include understanding how specific molecules are selected for inclusion in EVs released by donor cells, the regulatory mechanisms that control EV production, and the processes governing EV uptake and recycling in recipient cells. Additionally, clinical studies are essential to elucidate the clinical relevance of this non-genetic mode of MDR transmission, its impact on patient response to chemotherapy, and overall clinical outcome. Addressing these gaps will be critical for advancing our understanding and improving treatment outcomes in cancer therapy.

## DECLARATIONS

### Copyright

© The Author(s) 2024.
